# Comparison of Outcomes of Abdominal Wall Reconstruction Performed by Surgical Fellows vs Faculty

**DOI:** 10.1001/jamanetworkopen.2022.12444

**Published:** 2022-05-17

**Authors:** Abbas M. Hassan, Malke Asaad, Nikhil R. Shah, Francesco M. Egro, Jun Liu, Renata S. Maricevich, Jesse C. Selber, Matthew M. Hanasono, Charles E. Butler

**Affiliations:** 1 Department of Plastic and Reconstructive Surgery, The University of Texas MD Anderson Cancer Center, Houston; 2 Department of Plastic Surgery, University of Pittsburgh Medical Center, Pittsburgh, Pennsylvania; 3 Department of Surgery, The University of Texas Medical Branch, Galveston; 4 Department of Plastic Surgery, Baylor College of Medicine, Houston, Texas

## Abstract

**Importance:**

Concern regarding surgical trainees’ operative autonomy has increased in recent years, emphasizing patient safety and preparation for independent practice. Regarding abdominal wall reconstruction (AWR), long-term outcomes of fellow autonomy have yet to be delineated.

**Objectives:**

To evaluate the long-term outcomes of AWRs performed by fellows and compare them with those of AWRs performed by assistant, associate, and senior-level professors.

**Design, Setting, and Participants:**

This retrospective cohort study included patients who underwent AWR for ventral hernias or repair of tumor resection defects at a 710-bed tertiary cancer center between March 1, 2005, and June 30, 2019. The analysis was conducted between January 2020 and December 2021.

**Exposure:**

Academic rank of primary surgeon.

**Main Outcomes and Measures:**

The primary outcome was hernia recurrence. Secondary outcomes were surgical site occurrence, surgical site infection, length of hospital stay, unplanned return to the operating room, and 30-day readmission. Multivariable hierarchical models were constructed to identify predictive factors.

**Results:**

Of 810 consecutive patients, 720 (mean [SD] age, 59.8 [11.5] years; 375 female [52.1%]) met the inclusion criteria. Mean (SD) body mass index was 31.4 (6.7), and mean (SD) follow-up time was 42 (29) months. Assistant professors performed the most AWRs (276 [38.3%]), followed by associate professors (169 [23.5%]), senior-level professors (157 [21.8%]), and microsurgical fellows (118 [16.4%]). Compared with fellows and more junior surgeons, senior-level professors tended to operate on significantly older patients (mean [SD] age, 59.9 [10.9] years; *P* = .03), more patients with obesity (103 [65.6%]; *P* = .003), and patients with larger defects (247.9 [216.0] cm; *P* < .001), parastomal hernias (27 [17.2%]; *P* = .001), or rectus muscle violation (53 [33.8%]; *P* = .03). No significant differences were found for hernia recurrence, surgical site occurrence, surgical site infection, 30-day readmission rates, or length of stay among the fellows and assistant, associate, and senior-level professors in adjusted models. Compared with fellows, assistant professors (OR, 0.22; 95% CI, 0.08-0.64) and senior-level professors (OR, 0.20; 95% CI, 0.06-0.69) had lower rates of unplanned return to the operating room.

**Conclusions and Relevance:**

This cohort study provides evidence-based reassurance that providing fellows with autonomy in performing AWRs does not compromise long-term patient outcomes. These findings may incite efforts to increase appropriate surgical trainee autonomy, thereby empowering future generations of competent, independent surgeons.

## Key Points

**Question:**

Are the outcomes of abdominal wall reconstructions performed by surgical fellows comparable to those of reconstructions performed by faculty?

**Findings:**

In this cohort study of 720 consecutive abdominal wall reconstructions, operations performed by microsurgical fellows resulted in similar adjusted long-term hernia recurrence, surgical site occurrence, surgical site infection, 30-day readmission rates, and lengths of hospital stay compared with those performed by faculty, albeit with higher odds of unplanned return to the operating room.

**Meaning:**

These results suggest that providing fellows with operative autonomy does not compromise long-term outcomes of abdominal wall reconstructions; therefore, reversing the trend of diminishing operative autonomy in this domain is warranted to empower future generations of competent, independent surgeons.

## Teaser

This cohort study compares outcomes of abdominal wall reconstruction performed by surgical fellows with those of procedures performed by assistant, associate, and senior-level professors.

## Introduction

Commitment to training the next generation of surgeons while ensuring optimal patient safety is a challenging balancing act for residency and fellowship programs. The training paradigm has traditionally entailed a process of progressive, independent patient care delivery accompanied by a progressive reduction in supervision by attending surgeons, known as graduated responsibility. The trainee eventually achieves competency for independent practice, generally at or before completing the training program^1^; however, during the past decade, numerous changes in the surgical education model have threatened trainee education and the development of autonomy in the operating room.^2^ These changes include resident work hour regulations imposed by the Accreditation Council for Graduate Medical Education (ACGME), patient safety advocacy prompted by the Institute of Medicine, and Medicare and managed health care system policies.^3,4,5,6,7,8,9,10^ As a result of these changes, faculty supervision of surgical trainees has increased, whereas the degree of autonomy granted to trainees has significantly decreased, potentially altering the quality of graduating surgeons and impeding the advancement of surgical science. According to fellowship program directors, these changes have also resulted in residents who are undertrained, unprepared, and lacking in patient ownership.^11^

These changes have prompted critical analysis of the association between trainee autonomy and patient outcomes. Despite increased operative times and reports of increased risk of postoperative complications, a large and increasing body of literature in this area suggests that progressive surgical trainee autonomy is safe^12,13,14,15,16,17,18,19^; however, the studies reported in the literature evaluated large, heterogeneous patient cohorts for short follow-up periods. Furthermore, the researchers did not provide robust data on the degree of trainee autonomy and did not control for case complexity using finite preoperative, perioperative, and postoperative variables.

The long-term outcomes of surgical trainee autonomy have yet to be investigated in patients undergoing complex abdominal wall reconstruction (AWR), who have significant perioperative management considerations. This patient population is distinguished by a complex medical and surgical history and is thus at increased risk for postoperative complications.^20,21,22,23,24^ In addition, successful outcomes of AWR have been closely linked with the surgical technique used, highlighting the importance of adhering to appropriate evidence-based procedural algorithms to minimize complications.^22,25,26^ Therefore, one may infer that surgical resection and reconstruction performed by less experienced surgeons could pose additional risks to these patients; however, contextualizing the association between surgical trainee autonomy and long-term outcomes is of great interest to surgeons, trainees, patients, and health care policy makers to inform practices that promote trainee independence without compromising patient care.

In the current study, we evaluated the long-term outcomes of AWR performed by microsurgical fellows and compared them with those of AWR performed by assistant professors, associate professors, and senior-level professors. On the basis of our extensive clinical experience and comprehensive review of the literature, we hypothesized that AWR performed independently by fellows is safe, with durable long-term outcomes.

## Methods

### Study Design and Inclusion Criteria

This retrospective cohort study was conducted to assess consecutive patients who underwent open AWR to repair ventral hernias or resection of oncologic defects at The University of Texas MD Anderson Cancer Center from March 1, 2005, to June 30, 2019. The University of Texas MD Anderson Institutional Review Board approved this study and waived the need for informed consent. All data were deidentified. The study followed the Strengthening the Reporting of Observational Studies in Epidemiology (STROBE) reporting guideline.

Patients underwent midline AWR with placement of an underlay (preperitoneal, intraperitoneal, or retrorectus) acellular dermal matrix. Exclusion criteria for our study consisted of lateral abdominal wall hernia, resection defects lateral to the semilunar line, use of a posterior component separation technique (such as transversus abdominis release), onlay mesh placement, AWR with primary fascial closure without mesh, and a follow-up time shorter than 6 months. The surgical technique used in this study was consistent across all patients as described previously.^27,28,29^ Postoperative follow-up visits occurred weekly during the month after surgery, every 3 months for the next 6 months, and then yearly afterward.

Microsurgical fellows were trainees who had completed a full plastic surgery residency and were undergoing a 1-year microsurgery fellowship. Most ACGME-accredited plastic surgery residency programs commonly do not have robust AWR training; therefore, the fellows included in this study were mostly naive to AWR techniques. Regardless of their previous experience with AWR, fellows were generally trained on the AWR techniques consistently performed at The University of Texas MD Anderson Cancer Center by the senior faculty.^27,28,29^ Our fellowship program provides full credentialing for nonmicrosurgical procedures equivalent to a junior attending physician. Trainees have operative autonomy and complete independence in preoperative, intraoperative, and postoperative care. This autonomy includes decision-making, planning, operating, and management of complications (including additional operations); attending surgeon’s advice or assistance can be sought at any time, but the care remains driven by the fellows. In addition, fellows and faculty surgeons are assigned patients based on availability rather than by surgeon academic level or according to a referral pattern, reducing the risk of selection bias. Therefore, the current study evaluates surgical fellows who have proven independent autonomy, allowing for a credible comparison with more senior surgeons.

### Patient Selection

The patient demographic characteristics, comorbidities, defect characteristics, mesh characteristics, surgical outcomes, and complications were assessed using data from a prospectively maintained departmental database and electronic medical records. Patient race and ethnicity were omitted to protect patient confidentiality. Surgeons were classified based on academic rank as fellows, assistant professors, associate professors, or senior-level professors.

### Outcome Measures

The primary outcome of this study was the hernia recurrence rate. The secondary outcomes included surgical site occurrence (SSO), surgical site infection (SSI), 30-day readmission, and return to operating room rates and length of hospital stay. Hernia recurrence was defined as a contour abnormality with associated fascial defect diagnosed via physical examination and/or abdominal imaging with computed tomography or magnetic resonance imaging. An SSO was defined as skin necrosis, fat necrosis, wound dehiscence, infection, hematoma, seroma, or enterocutaneous fistula. Seroma and hematoma were defined as serous fluid and blood collections, respectively, requiring percutaneous or operative drainage. Wound dehiscence was defined as a breakdown of the incision with full-thickness skin separation in the presence or absence of infection that extended more than 2 cm. Fat necrosis was defined as a greater than 1-cm palpable firmness that persisted for longer than 3 months postoperatively. Surgical site infections consisted of infectious processes, either abscesses or cellulitis, requiring treatment with antibiotics with or without drainage. Rectus muscle violation was defined as an existing or new ostomy, gastrostomy or jejunostomy tube placement, transversely divided rectus abdominis muscle, and/or resected rectus abdominis muscle. Obesity was defined as a body mass index (calculated as weight in kilograms divided by height in meters squared) of at least 30. Bridged repair was defined as the use of mesh to span a defect in the abdominal wall without approximating the fascial edges.

### Statistical Analysis

Descriptive statistics were used to summarize continuous variables, and numbers (percentages) were used for categorical variables. A χ^2^ test or the Fisher exact test was used to compare categorical variables among study groups. Analysis of variance or the Kruskal-Wallis test was used to examine differences in continuous variables. The time to hernia recurrence was defined as the interval from the date of AWR to the date of hernia recurrence or last follow-up visit, whichever occurred first. Patients who did not develop hernia recurrence at the last follow-up date were censored in the analysis. Cumulative hernia recurrence–free probabilities were estimated using the Kaplan-Meier product-limit method. A log-rank test was used to compare the distribution of hernia recurrence–free probability among various patient groups. Multilevel models were designed with patients nested within surgeons. A multivariable, hierarchical, Cox frailty proportional hazards regression model was used to evaluate the effect of academic rank on time to hernia recurrence. Multivariable hierarchical logistic regression models were used to assess the association of surgeon academic rank with SSO, SSI, unplanned return to the operating room, and 30-day readmission rate. A multivariable hierarchical mixed linear regression model was used to evaluate the association of academic rank with length of hospital stay. The logarithm transformation of the length of stay was applied for a better model fit. Parsimonious multivariable models were fitted by stepwise model selection. Covariates included patient age, sex, body mass index, comorbidities, tobacco use, receipt of chemotherapy or radiation therapy, wound contamination class, American Society of Anesthesiologists class, presence of rectus muscle violation or parastomal hernia, prior abdominal operations or hernia repair, indications for surgery, defect and mesh characteristics (width, length, and size), and surgical techniques (component separation, bridged repair, and panniculectomy). Missing values were imputed using the normal, Bernoulli, and multinomial distribution for continuous, binary, and categorical variables, respectively. A sensitivity analysis was performed by comparing the models that were performed in the complete set and the imputed data set. Two-tailed *P* < .05 was considered significant, and all statistical analyses were conducted between January 2020 and December 2021 with SAS Enterprise Guide software, version 9.4 (SAS Institute Inc) by a senior biostatistician (J.L.).

## Results

### Patient Demographic Characteristics

Of 810 consecutive patients, we identified 720 (mean [SD] age, 59.8 [11.5] years; 375 female [52%] and 345 male [48%]) who met the inclusion criteria. The mean (SD) body mass index was 31.4 (6.7), and the mean (SD) follow-up time was 42 (29) months. Assistant professors performed the most AWRs (276 [38.3%]), followed by associate professors (169 [23.5%]), senior-level professors (157 [21.8%]), and fellows (118 [16.4%]). No attending physicians assisted in any of the 118 cases performed by fellows. The patients’ demographic and surgical characteristics varied by the academic rank of the surgeons (eTables 1 and 2 in the Supplement). Compared with the fellows and more junior professors, senior-level professors operated on significantly older patients (mean [SD] age, 59.9 [10.9] years; *P* = .03), more patients with obesity (103 [65.6%]; *P* = .003), and patients with larger defects (mean [SD] defect size, 247.9 [216.0] cm; *P* < .001), parastomal hernias (27 [17.2%]; *P* = .001), or rectus muscle violation (53 [33.8%]; *P* = .03). Many of our patients had an American Society of Anesthesiologists physical status classification of III or IV (623 [86.5%]), were obese (401 [55.7%]), had contaminated or infected defects (99 [13.8%]), and had a mean (SD) defect width of 180.1 (173.4) cm.

### Hernia Recurrence

Physicians performed postoperative computed tomography for 673 patients (93.5%) in accordance with disease-specific oncologic surveillance or if hernia recurrence was suspected on physical examination. Hernia recurrence was identified in 93 patients (12.9%). In an adjusted multivariable, hierarchical, Cox frailty proportional hazards regression model (Table 1), we found no significant differences in hernia recurrence rates among assistant professors (hazard ratio [HR], 1.01; 95% CI, 0.51-1.96), associate professors (HR, 1.82; 95% CI, 0.81-4.08), or senior-level professors (HR, 2.07; 95% CI, 0.91-4.66) compared with fellows.

**Table 1.** Univariate and Multivariable Hierarchical Cox Frailty Proportional Hazards Regression Models of Hernia Recurrence

Variable	Univariate model		Multivariable model	
	HR (95% CI)	*P* value	HR (95% CI)	*P* value
Surgeon’s academic rank				
Fellow	1 [Reference]	NA	1 [Reference]	NA
Assistant professor	0.73 (0.38-1.40)	.34	1.01 (0.51-1.96)	.98
Associate professor	1.03 (0.50-2.10)	.95	1.82 (0.81-4.08)	.15
Senior-level professor	1.58 (0.79-3.16)	.20	2.07 (0.91-4.66)	.08
Wound classification				
Clean	1 [Reference]	NA	NA	NA
Clean-contaminated, contaminated, or infected	1.45 (0.95-2.22)	.09	NA	NA
Age	0.99 (0.97-1.01)	.22	NA	NA
Male sex	1.06 (0.70-1.59)	.79	NA	NA
Body mass index	1.04 (1.01-1.07)	.007	NA	NA
Obesity	2.25 (1.42-3.54)	<.001	2.46 (1.55-3.91)	<.001
Tobacco use	1.10 (0.53-2.28)	.81	NA	NA
Any comorbidity	0.99 (0.65-1.51)	.95	NA	NA
Coronary artery disease	1.36 (0.72-2.57)	.34	NA	NA
Diabetes	1.41 (0.88-2.27)	.16	NA	NA
Pulmonary disease	1.36 (0.70-2.63)	.36	NA	NA
Kidney disease	1.54 (0.83-2.85)	.17	NA	NA
Preoperative radiotherapy	1.35 (0.87-2.10)	.18	NA	NA
Preoperative chemotherapy	1.26 (0.82-1.95)	.30	NA	NA
ASA status				
2	1 [Reference]	NA	NA	NA
3 or 4	1.15 (0.59-2.23)	.68	NA	NA
Rectus muscle violation	1.72 (1.12-2.65)	.01	1.72 (1.12-2.64)	.01
Parastomal hernia	1.44 (0.79-2.62)	.23	NA	NA
Prior abdominal surgery (yes vs no)	0.95 (0.30-3.04)	.94	NA	NA
Prior hernia repair (yes vs no)	1.44 (0.93-2.24)	.10	NA	NA
Indication for AWR (primary hernia vs extirpative defect)	1.09 (0.68-1.76)	.72	NA	NA
Defect width, by 5 cm	1.00 (0.88-1.13)	.97	NA	NA
Defect length, by 5 cm	1.15 (0.97-1.35)	.10	NA	NA
Defect size, by 50 cm^2^	1.06 (1.00-1.13)	.06	1.03 (0.97-1.08)	.38
Defect width >15 cm	0.90 (0.57-1.42)	.64	NA	NA
Component separation (yes vs no)	0.44 (0.29-0.68)	<.001	0.43 (0.27-0.66)	<.001
Bridged repair	3.82 (2.37-6.16)	<.001	3.15 (1.92-5.20)	<.001
Panniculectomy	0.98 (0.61-1.59)	.94	NA	NA
Mesh width, by 5 cm	1.19 (0.95-1.49)	.13	NA	NA
Mesh length, by 5 cm	1.06 (0.90-1.24)	.48	NA	NA
Mesh size, by 50 cm^2^	1.04 (0.99-1.09)	.11	NA	NA

Abbreviations: ASA, American Society of Anesthesiologists; AWR, abdominal wall reconstruction; HR, hazard ratio; NA, not applicable.

### Surgical Site Occurrences

A total of 219 patients (30.4%) had SSOs, with wound dehiscence being the most common (110 [15.3%]). In an adjusted multivariable hierarchical logistic regression model (Table 2), we found no significant differences in SSO rate among assistant professors (odds ratio [OR], 1.25; 95% CI, 0.70-2.24), associate professors (OR, 1.66; 95% CI, 0.81-3.41), or senior-level professors (OR, 1.38; 95% CI, 0.65-2.92) compared with fellows.

**Table 2.** Univariate and Multivariable Hierarchal Logistic Regression Models of Surgical Site Occurrence

Variable	Univariate model		Multivariable model	
	OR (95% CI)	*P* value	OR (95% CI)	*P* value
Surgeon’s academic rank				
Fellow	1 [Reference]	NA	1 [Reference]	NA
Assistant professor	1.09 (0.64-1.84)	.75	1.25 (0.70-2.24)	.45
Associate professor	1.05 (0.60-1.86)	.85	1.66 (0.81-3.41)	.17
Senior-level professor	1.11 (0.59-2.07)	.74	1.38 (0.65-2.92)	.41
Wound classification				
Clean	1 [Reference]	NA	NA	NA
Clean-contaminated, contaminated, or infected	1.95 (1.37-2.79)	<.001	NA	NA
Age	0.99 (0.98-1.01)	.44	NA	NA
Male sex	0.76 (0.54-1.06)	.11	0.64 (0.45-0.93)	.02
Body mass index	1.07 (1.04-1.09)	<.001	1.05 (1.03-1.08)	<.001
Obesity	2.22 (1.56-3.17)	<.001	NA	NA
Tobacco use	1.03 (0.55-1.93)	.91	NA	NA
Any comorbidity	1.83 (1.26-2.65)	.001	1.54 (1.04-2.28)	.03
Coronary artery disease	1.25 (0.70-2.21)	.45	NA	NA
Diabetes	1.94 (1.32-2.86)	.001	NA	NA
Pulmonary disease	1.88 (1.11-3.18)	.02	NA	NA
Kidney disease	1.50 (0.86-2.64)	.16	NA	NA
Preoperative radiotherapy	1.18 (0.81-1.72)	.40	NA	NA
Preoperative chemotherapy	0.79 (0.56-1.11)	.18	NA	NA
ASA status				
2	1 [Reference]	NA	NA	NA
3 or 4	1.59 (0.88-2.89)	.12	NA	NA
Rectus muscle violation	2.00 (1.39-2.87)	<.001	2.14 (1.44-3.17)	<.001
Parastomal hernia	1.33 (0.77-2.29)	.30	NA	NA
Prior abdominal surgery (yes vs no)	1.22 (0.48-3.11)	.68	NA	NA
Prior hernia repair (yes vs no)	1.21 (0.83-1.76)	.33	NA	NA
Indication for AWR (primary hernia vs extirpative defect)	0.80 (0.55-1.16)	.23	NA	NA
Defect width, by 5 cm	1.12 (1.02-1.23)	.02	NA	NA
Defect length, by 5 cm	1.21 (1.06-1.38)	.004	NA	NA
Defect size, by 50 cm^2^	1.07 (1.02-1.12)	.004	1.01 (0.96-1.07)	.60
Defect width >15 cm	1.21 (0.85-1.74)	.29	NA	NA
Component separation (yes vs no)	1.42 (0.99-2.04)	.05	1.44 (0.97-2.12)	.07
Bridged repair	1.97 (1.21-3.21)	.007	2.04 (1.19-3.50)	.009
Panniculectomy	1.28 (0.89-1.83)	.18	NA	NA
Mesh width, by 5 cm	1.44 (1.19-1.74)	<.001	NA	NA
Mesh length, by 5 cm	1.35 (1.18-1.54)	<.001	NA	NA
Mesh size, by 50 cm^2^	1.10 (1.06-1.15)	<.001	1.07 (1.02-1.12)	.004

Abbreviations: ASA, American Society of Anesthesiologists; AWR, abdominal wall reconstruction; NA, not applicable; OR, odds ratio.

### Surgical Site Infections

A total of 92 patients (12.8%) experienced SSIs. In an adjusted multivariable, hierarchical logistic regression model (Table 3), we found no significant differences in SSI rate among assistant professors (OR, 0.92; 95% CI, 0.45-1.90), associate professors (OR, 0.88; 95% CI, 0.35-2.22), or senior-level professors (OR, 1.10; 95% CI, 0.43-2.80) compared with fellows.

**Table 3.** Univariate and Multivariable Hierarchical Logistic Regression Models of Surgical Site Infection

Variable	Univariate model		Multivariable model	
	OR (95% CI)	*P* value	OR (95% CI)	*P* value
Surgeon’s academic rank				
Fellow	1 [Reference]	NA	1 [Reference]	NA
Assistant professor	0.78 (0.41-1.47)	.44	0.92 (0.45-1.90)	.83
Associate professor	0.71 (0.35-1.44)	.34	0.88 (0.35-2.22)	.79
Senior-level professor	1.13 (0.58-2.20)	.73	1.10 (0.43-2.80)	.85
Wound classification				
Clean	1 [Reference]	NA	NA	NA
Clean-contaminated, contaminated, or infected	1.39 (0.88-2.21)	.16	NA	NA
Age	1.01 (0.99-1.03)	.53	NA	NA
Male sex	0.86 (0.55-1.33)	.49	NA	NA
Body mass index	1.03 (1.00-1.06)	.05	NA	NA
Obesity	1.76 (1.11-2.80)	.02	1.52 (0.94-2.47)	.09
Tobacco use	1.20 (0.55-2.65)	.64	NA	NA
Any comorbidity	1.51 (0.92-2.46)	.10	NA	NA
Coronary artery disease	1.54 (0.77-3.07)	.22	NA	NA
Diabetes	1.25 (0.75-2.10)	.40	NA	NA
Pulmonary disease	1.75 (0.91-3.35)	.09	NA	NA
Kidney disease	1.99 (1.03-3.87)	.04	NA	NA
Preoperative radiotherapy	0.89 (0.53-1.49)	.66	NA	NA
Preoperative chemotherapy	0.69 (0.45-1.08)	.11	0.62 (0.39-0.99)	.04
ASA status				
2	1 [Reference]	NA	NA	NA
3 or 4	1.31 (0.61-2.82)	.50	NA	NA
Rectus muscle violation	2.08 (1.32-3.30)	.002	2.10 (1.29-3.40)	.003
Parastomal hernia	1.53 (0.79-2.99)	.21	NA	NA
Prior abdominal surgery (yes vs no)	3.77 (0.50-28.3)	.20	NA	NA
Prior hernia repair (yes vs no)	1.31 (0.81-2.13)	.27	NA	NA
Indication for AWR (primary hernia vs extirpative defect)	0.87 (0.54-1.41)	.58	NA	NA
Defect width, by 5 cm	1.17 (1.04-1.32)	.01	NA	NA
Defect length, by 5 cm	1.23 (1.04-1.45)	.01	NA	NA
Defect size, by 50 cm^2^	1.08 (1.02-1.14)	.01	1.03 (0.97-1.10)	.33
Defect width >15 cm	1.41 (0.89-2.21)	.14	NA	NA
Component separation (yes vs no)	1.03 (0.65-1.63)	.89	NA	NA
Bridged repair	2.03 (1.13-3.66)	.02	NA	NA
Panniculectomy	1.09 (0.69-1.72)	.70	NA	NA
Mesh width, by 5 cm	1.31 (1.04-1.64)	.02	NA	NA
Mesh length, by 5 cm	1.37 (1.16-1.61)	<.001	0.82 (0.68-0.98)	.03
Mesh size, by 50 cm^2^	1.09 (1.04-1.14)	.001	1.06 (1.01-1.12)	.02

Abbreviations: ASA, American Society of Anesthesiologists; AWR, abdominal wall reconstruction; NA, not applicable; OR, odds ratio.

### Length of Hospital Stay

The mean (SD) length of hospital stay was 9.1 (15.7) days. In an adjusted multivariable, hierarchical mixed linear regression model (Table 4), we found no significant differences in length of hospital stay among assistant professors (β = 0.04; 95% CI, −0.10 to 0.17), associate professors (β = 0.06; 95% CI, −0.11 to 0.24), or senior-level professors (β = 0.07; 95% CI, −0.13 to 0.27) compared with fellows.

**Table 4.** Univariate and Multivariable Hierarchical Mixed Linear Regression Models of Length of Hospital Stay

Variable	Univariate model		Multivariable model	
	β (95% CI)	*P* value	β (95% CI)	*P* value
Surgeon’s academic rank				
Fellow	1 [Reference]	NA	1 [Reference]	NA
Assistant professor	−0.02 (−0.16 to 0.12)	.78	0.04 (−0.10 to 0.17)	.60
Associate professor	−0.11 (−0.27 to 0.05)	.17	0.06 (−0.11 to 0.24)	.48
Senior-level professor	−0.25 (−0.42 to −0.07)	.006	0.07 (−0.13 to 0.27)	.47
Wound classification				
Clean	1 [Reference]	NA	NA	NA
Clean-contaminated, contaminated, or infected	0.42 (0.34 to 0.49)	<.001	0.36 (0.27 to 0.45)	<.001
Age	0.005 (0.002 to 0.009)	.006	0.005 (0.002 to 0.009)	.003
Male sex	0.17 (0.09 to 0.25)	<.001	0.09 (0.01 to 0.17)	.03
Body mass index	0.003 (−0.004 to 0.01)	.83	NA	NA
Obesity	−0.01 (−0.09 to 0.07)	.77	NA	NA
Tobacco use	0.15 (−0.00 to 0.30)	.05	NA	NA
Any comorbidity	0.09 (0.01 to 0.18)	.04	NA	NA
Coronary artery disease	0.21 (0.06 to 0.35)	.005	NA	NA
Diabetes	0.06 (−0.05 to 0.16)	.28	NA	NA
Pulmonary disease	0.12 (−0.02 to 0.26)	.10	NA	NA
Kidney disease	0.23 (0.08 to 0.38)	.003	NA	NA
Preoperative radiotherapy	0.05 (−0.04 to 0.14)	.29	NA	NA
Preoperative chemotherapy	−0.01 (−0.09 to 0.08)	.90	NA	NA
ASA status				
2	1 [Reference]	NA	NA	NA
3 or 4	0.20 (0.07 to 0.33)	.003	NA	NA
Rectus muscle violation	0.28 (0.19 to 0.38)	<.001	0.19 (0.07 to 0.30)	.001
Parastomal hernia	0.08 (−0.06 to 0.22)	.26	−0.22 (−0.38 to −0.05)	.009
Prior abdominal surgery (yes vs no)	0.10 (−0.11 to 0.32)	.35	NA	NA
Prior hernia repair (yes vs no)	0.06 (−0.04 to 0.15)	.24	NA	NA
Indication for AWR (primary hernia vs extirpative defect)	−0.19 (−0.28 to −0.09)	<.001	−0.20 (−0.30 to −0.11)	<.001
Defect width, by 5 cm	0.05 (0.03 to 0.08)	<.001	0.03 (0.00 to 0.05)	.03
Defect length, by 5 cm	0.07 (0.03 to 0.10)	<.001	NA	NA
Defect size, by 50 cm^2^	0.03 (0.02 to 0.04)	<.001	NA	NA
Defect width >15 cm	0.15 (0.05 to 0.24)	.002	NA	NA
Component separation (yes vs no)	0.03 (−0.05 to 0.12)	.44	NA	NA
Bridged repair	0.24 (0.11 to 0.37)	<.001	0.20 (0.07 to 0.33)	.003
Panniculectomy	−0.01 (−0.10 to 0.09)	.90	NA	NA
Mesh width, by 5 cm	0.14 (0.09 to 0.18)	<.001	NA	NA
Mesh length, by 5 cm	0.11 (0.08 to 0.14)	<.001	NA	NA
Mesh size, by 50 cm^2^	0.04 (0.03 to 0.05)	<.001	0.03 (0.02 to 0.04)	<.001

Abbreviations: ASA, American Society of Anesthesiologists; AWR, abdominal wall reconstruction; NA, not applicable.

### Unplanned Return to the Operating Room

A total of 72 patients (10.0%) had unplanned returns to the operating room. In an adjusted multivariable hierarchical logistic regression model (Table 5), assistant professors (OR, 0.22; 95% CI, 0.08-0.64) and senior-level professors (OR, 0.20; 95% CI, 0.06-0.69) had lower rates of unplanned return to the operating room than did fellows. eTable 3 in the Supplement gives the specific causes of the return to the operating room for each of the cases, including the indication for the AWR, the timing of the subsequent operation, the reason for the subsequent operation, and whether an attending physician assisted in the surgery. Thirteen of the AWRs performed by fellows (11.0%) required a return to the operating room. Five (38.5%) of the subsequent operations occurred within 30 days, 9 (69.2%) within 6 months, and 4 (30.8%) after 6 months of follow-up. A total of 11 subsequent operations (84.6%) were due to either an SSO or an SSI, and the most common reason (7 [53.8%]) was incision and drainage of an abscess. Of the 9 subsequent operations that occurred during the fellowship timeframe, 6 (66.6%) were performed independently by the fellows.

**Table 5.** Univariate and Multivariable Hierarchical Logistic Regression Models of Unplanned Return to the Operating Room

Variable	Univariate model		Multivariable model	
	OR (95% CI)	*P* value	OR (95% CI)	*P* value
Surgeon’s academic rank				
Fellow	1 [Reference]	NA	1 [Reference]	NA
Assistant professor	0.57 (0.28-1.20)	.14	0.22 (0.08-0.64)	.005
Associate professor	1.21 (0.58-2.53)	.60	0.42 (0.13-1.33)	.14
Senior-level professor	0.73 (0.33-1.65)	.45	0.20 (0.06-0.69)	.01
Wound classification				
Clean	1 [Reference]	NA	NA	NA
Clean-contaminated, contaminated, or infected	1.31 (0.78-2.17)	.31	NA	NA
Age, y	0.99 (0.97-1.01)	.22	NA	NA
Sex, male	1.42 (0.87-2.32)	.17	NA	NA
Body mass index	1.02 (0.99-1.06)	.21	NA	NA
Obesity	1.02 (0.62-1.67)	.95	NA	NA
Tobacco use	0.70 (0.24-2.01)	.51	NA	NA
Any comorbidity	1.26 (0.74-2.14)	.39	NA	NA
Coronary artery disease	0.78 (0.30-2.03)	.61	NA	NA
Diabetes	1.14 (0.63-2.07)	.66	NA	NA
Pulmonary disease	1.71 (0.83-3.56)	.15	NA	NA
Kidney disease	1.39 (0.63-3.08)	.41	NA	NA
Preoperative radiotherapy	0.98 (0.55-1.74)	.95	NA	NA
Preoperative chemotherapy	1.26 (0.74-2.13)	.39	NA	NA
ASA status				
2	1 [Reference]	NA	NA	NA
3 or 4	1.00 (0.45-2.19)	.99	NA	NA
Rectus muscle violation	2.08 (1.24-3.49)	.006	1.99 (1.16-3.39)	.01
Parastomal hernia	1.48 (0.69-3.17)	.31	NA	NA
Prior abdominal surgery (yes vs no)	1.34 (0.31-5.85)	.70	NA	NA
Prior hernia repair (yes vs no)	1.30 (0.75-2.23)	.35	NA	NA
Indication for AWR (primary hernia vs extirpative defect)	0.83 (0.48-1.41)	.48	NA	NA
Defect width, by 5 cm	0.95 (0.82-1.11)	.52	NA	NA
Defect length, by 5 cm	1.06 (0.87-1.30)	.55	NA	NA
Defect size, by 50 cm^2^	1.00 (0.93-1.08)	.99	NA	NA
Defect width >15 cm	0.70 (0.39-1.25)	.23	NA	NA
Component separation (yes vs no)	0.86 (0.51-1.45)	.58	NA	NA
Bridged repair	1.71 (0.87-3.38)	.12	NA	NA
Panniculectomy	0.92 (0.53-1.58)	.76	NA	NA
Mesh width, by 5 cm	1.16 (0.89-1.51)	.28	NA	NA
Mesh length, by 5 cm	1.30 (1.09-1.56)	.003	NA	NA
Mesh size, by 50 cm^2^	1.09 (1.03-1.15)	.003	1.08 (1.02-1.15)	.005

Abbreviations: ASA, American Society of Anesthesiologists; AWR, abdominal wall reconstruction; NA, not applicable; OR, odds ratio.

### 30-Day Readmission

A total of 78 patients (10.8%) were readmitted within 30 days after AWR. In an adjusted multivariable hierarchical logistic regression model (eTable 4 in the Supplement), we found no significant differences in 30-day readmission rate among assistant professors (OR, 0.62; 95% CI, 0.26-1.46), associate professors (OR, 0.64; 95% CI, 0.22-1.85), or senior-level professors (OR, 0.41; 95% CI, 0.13-1.27) compared with fellows.

## Discussion

In this cohort study, we sought to illuminate the long-term outcomes of surgical fellow autonomy in patients undergoing complex AWR during a mean follow-up time of 42 months. We found that senior-level professors operated on older patients with more complex defects than did other surgeons. However, after adjusting for relevant risk factors and case complexity, we found no differences in long-term hernia recurrence, SSO, SSI, 30-day readmission rates, or length of hospital stay between fellows and more senior surgeons. We found greater odds of unplanned return to the operating room in the fellows’ cases than in the other cases. With a national trend of reducing operative autonomy in light of patient safety concerns, these findings provide timely, discrete evidence to reassure attending surgeons and advocate for a shift toward increased trainee autonomy.

These findings are consistent with a large body of literature^12,13,14,15,16,17,18,19^ that has documented similar short-term outcomes of operations performed by residents and those performed entirely by attending surgeons. In a recent study, Oliver et al^12^ analyzed data from the Veterans Affairs Surgical Quality Improvement Program to evaluate the association between resident autonomy and surgical outcomes. They found no significant differences in length of hospital stay, all-cause morbidity, or patient mortality when comparing surgical procedures performed by residents alone and those performed by attending surgeons alone or with residents. Similar results were reported using the National Surgical Quality Improvement Program database. Lakomkin and Hadjipanayis^19^ demonstrated that resident involvement was not significantly associated with mortality, length of hospital stay, readmission, subsequent operation, or surgical complications after brain tumor resection.

Although the findings of these studies are encouraging, the use of the Veterans Affairs Surgical Quality Improvement Program and National Surgical Quality Improvement Program databases pose several inherent limitations. First, resident involvement was self-reported by surgeons with compromised granularity and the use of surrogates for operative autonomy. Second, the reported data were limited to short-term, 30-day complications, with no assessment of critical long-term outcomes. Third, patient-level data and surgical complexity are not captured in such databases, limiting the ability to adjust for competing risk factors and case complexity. With our study design, we addressed these limitations and leveraged 14 years of cumulative experience at a major cancer center during a mean (SD) follow-up time of 42 (29) months while adjusting for relevant risk factors and case complexity using multivariable hierarchical regression analysis.

Although Mattar et al^11^ reported on the readiness of surgical fellows for independence and unsupervised operating by surveying fellowship program directors, no study has yet evaluated the outcomes of patients operated on independently by surgical fellows. We demonstrated no difference in surgical outcomes between faculty and fellows, providing reassurance that fellows are ready for independence without compromising patient outcomes. These findings fill a critical knowledge gap and call into question the existing literature, which has suggested that graduates of surgical residency are not prepared for independence in fellowship. However, there remains a gap between the expectations of fellowship program directors and fellow outcomes (ie, despite having comparable outcomes to faculty, why are fellows still perceived as undertrained, unprepared, and lacking in patient ownership?).^11^ Future studies are warranted to more clearly elucidate the mechanisms responsible for the difference in fellowship director expectations and fellow outcomes.

Unlike SSOs and SSIs, which were not different between faculty and fellows, faculty surgeons had lower rates of unplanned return to the operating room than fellows. One explanation for these findings may be that patients who develop SSOs or SSIs are identified at a later stage by the fellows and are thus more likely to require a return to the operating room. It is also possible that patients operated on by fellows have more severe manifestation of surgical complications that necessitate a return to the operating room rather than earlier management in the clinic setting. However, one could argue that the fellows should not be faulted for returning to the operating room in surgically complex cases (eTable 3 in the Supplement) because such cases are already at a highly unpredictable risk of complications regardless of the surgeon’s academic rank. Our findings suggest that the knowledge and wisdom of experienced faculty may be invaluable for fellows in mitigating complications and return to the operating room in complex cases.

The patient population analyzed in this study was immunocompromised and had significant medical and surgical comorbidities that increased their risk of hernia recurrence and postoperative complications.^29,30,31,32,33^ Furthermore, many of our patients had an American Society of Anesthesiologists physical status classification of III or IV, were obese, had contaminated or infected defects, and had large defects. Despite our patient population’s complexity and heightened risk of complications, the adjusted long-term surgical outcomes were comparable for AWRs performed by fellows and those performed by senior surgeons. This result only strengthens our findings. Outcomes of fellows’ autonomy are expected to be better in the general population, which has a lower baseline surgical risk, particularly among nonimmunocompromised individuals.

Although our findings provide evidence-based reassurance to attending surgeons that giving fellows appropriate operative autonomy does not compromise patient outcomes, patient safety is not the only factor influencing the level of independence granted to residents and fellows. Concern regarding malpractice liabilities, ethical considerations, and pressure to increase productivity can potentially incentivize attending surgeons to limit trainee operative autonomy.^6,34^ However, we believe that early investment in fellow autonomy, leadership development, and decision-making during training should be an institutional commitment to ensure future favorable outcomes and well-being. Although beyond the scope of our study and the expertise of our research team, we hope that our results catalyze the ACGME, American Board of Surgery, and American College of Surgeons to increase appropriate trainee autonomy. Pursuant to this outcome, a system should be designed to address other factors that impede trainee autonomy to enhance the development of future surgeons.

### Limitations

Our study has certain limitations, including its single-institution, nonrandomized, retrospective design, which could have resulted in selection bias and confounding factors. Given the complexity of our patient population, our results may not be representative of those for patients in community or referral centers and hence may not be generalizable to those settings. In addition, the varying training history and technical skill levels of the surgical fellows were not quantifiable in this study and may have introduced unforeseen confounding factors based on their training programs. However, their training level was consistent in that all fellows had just completed a plastic surgery residency. Although we adjusted for various surgical factors, senior faculty may have operated on more complex cases with elements not captured in the medical records, potentially introducing selection bias. Our study demonstrated the long-term outcomes of fellow autonomy in patients undergoing AWR. However, because we only train full-time fellows at our institution, we did not address rotating resident autonomy, which may be a greater concern for patient safety. The best balance between patient safety and resident independence should be elucidated in future studies. Lastly, we did not include patient-reported outcome measures, which are important considerations in outcome evaluation. Because of these limitations, future randomized, prospective, multicenter studies are warranted. Notwithstanding these limitations, our study is the first, to our knowledge, to assess the long-term outcomes of surgical fellow autonomy while controlling for relevant risk factors and case complexity.

## Conclusions

In this cohort study of patients undergoing complex AWR, we found no differences in adjusted long-term hernia recurrence, SSO, SSI, or 30-day readmission rates or length of hospital stay between operations performed by fellows and those performed by faculty. However, we did find greater odds of unplanned return to the operating room in the fellows’ cases. These findings provide evidence-based reassurance to attending surgeons and patients that giving fellows operative autonomy does not compromise long-term patient outcomes. Our hope is that this evidence incites efforts to increase surgical trainee autonomy, thereby empowering future generations of competent, independent surgeons.

## Notes

Supplement. **eTable 1.** Patient Demographics **eTable 2.** Surgical Characteristics **eTable 3.** Reasons for Reoperation in the Cases Operated on by Fellows **eTable 4.** Univariate and Multivariable Hierarchical Logistic Regression Model of 30-Day Readmission
