# Supplementary material for: Comparison of Outcomes of Abdominal Wall Reconstruction Performed by Surgical Fellows vs Faculty
Source: JAMA Netw Open. 2022 May 17;5(5):e2212444. doi: 10.1001/jamanetworkopen.2022.12444 (PMC9115612; doi:10.1001/jamanetworkopen.2022.12444)
Supplement: Supplement. — eTable 1. Patient Demographics eTable 2. Surgical Characteristics eTable 3. Reasons for Reoperation in the Cases Operated on by Fellows eTable 4. Univariate and Multivariable Hierarchical Logistic Regression Model of 30-Day Readmission [file jamanetwopen-e2212444-s001.pdf]

Supplementary Online Content

Hassan AM, Asaad M, Shah NR, et al. Comparison of outcomes of abdominal wall reconstruction performed by surgical fellows vs faculty. *JAMA Netw Open*. 2022;5(5):e2212444. doi:10.1001/jamanetworkopen.2022.12444

eTable 1. Patient Demographics
eTable 2. Surgical Characteristics
eTable 3. Reasons for Reoperation in the Cases Operated on by Fellows
eTable 4. Univariate and Multivariable Hierarchical Logistic Regression Model of 30-Day Readmission

This supplementary material has been provided by the authors to give readers additional information about their work.

© 2022 Hassan AM et al. *JAMA Network Open.*

eTable 1. Patient Demographics

Variable	Fellow	Assistant Professor	Associate Professor	Professor	P value
Number of patients	118	276	169	157	
Age	57.55 +/- 12.75	59.55 +/- 11.86	61.72 +/- 10.33	59.86 +/- 10.93	0.026
Body mass index	32.32 +/- 6.93	31.25 +/- 6.87	30.49 +/- 6.34	31.99 +/- 6.23	0.027
Length of follow up (months)					0.099
Mean+/-SD	40.87 +/- 27.9	44.12 +/- 28.06	38.83 +/- 26.58	40.88 +/- 31.98	
Median (IQR)	33.19 (18.71, 54.12)	38.1 (21.4, 58.03)	31.74 (21.06, 50.58)	32.3 (14.84, 54.58)	
Gender					0.039
Female	61 (51.7)	158 (57.2)	73 (43.2)	83 (52.9)	
Male	57 (48.3)	118 (42.8)	96 (56.8)	74 (47.1)	
Obesity	72 (61)	147 (53.3)	79 (46.7)	103 (65.6)	0.003
Tobacco use	11 (9.3)	20 (7.2)	11 (6.5)	12 (7.6)	0.841
Any comorbidity	77 (65.3)	174 (63)	116 (68.6)	102 (65)	0.694
Coronary artery disease	13 (11)	24 (8.7)	14 (8.3)	11 (7)	0.704
Diabetes mellitus	21 (17.8)	48 (17.4)	45 (26.6)	34 (21.7)	0.103
Hypertension	65 (55.1)	148 (53.6)	87 (51.5)	89 (56.7)	0.811
Pulmonary disease	14 (11.9)	26 (9.4)	15 (8.9)	12 (7.6)	0.689
Renal disease	16 (13.6)	25 (9.1)	12 (7.1)	8 (5.1)	0.079
Preoperative abdominal radiotherapy	30 (25.4)	73 (26.4)	36 (21.3)	47 (29.9)	0.354

© 2022 Hassan AM et al. *JAMA Network Open.*

Postoperative abdominal wall radiotherapy	9 (7.6)	14 (5.1)	9 (5.3)	1 (0.6)	0.037
Preoperative chemotherapy	68 (57.6)	184 (66.7)	106 (62.7)	96 (61.1)	0.349
Intraoperative chemotherapy	2 (1.7)	9 (3.3)	6 (3.6)	5 (3.2)	0.819
Postoperative chemotherapy	38 (32.2)	57 (20.7)	33 (19.5)	37 (23.6)	0.053
ASA status					0.729
unknown	2 (1.7)	6 (2.2)	7 (4.1)	6 (3.8)	
2	10 (8.5)	32 (11.6)	20 (11.8)	14 (8.9)	
3	99 (83.9)	225 (81.5)	135 (79.9)	133 (84.7)	
4	7 (5.9)	13 (4.7)	7 (4.1)	4 (2.5)	
3/4	106 (89.8)	238 (86.2)	142 (84)	137 (87.3)	0.651
Would classification					0.410
Clean	45 (38.1)	105 (38)	75 (44.4)	74 (47.1)	
Clean-contaminated	56 (47.5)	128 (46.4)	73 (43.2)	65 (41.4)	
Contaminated	13 (11)	30 (10.9)	10 (5.9)	13 (8.3)	
Infected	4 (3.4)	13 (4.7)	11 (6.5)	5 (3.2)	

IQR, interquartile range; ASA, American Society of Anesthesiologists

© 2022 Hassan AM et al. *JAMA Network Open.*

eTable 2. Surgical Characteristics

Variable	Fellow	Assistant Professor	Associate Professor	Professor	P value
Defect Width	13.36 +/- 8.23	13.12 +/- 8.21	14.09 +/- 9.42	15.67 +/- 10.18	0.234
Defect size	167.04 +/- 166	146.24 +/- 137.61	180.78 +/- 169.29	247.87 +/- 215.95	<0.001
Mesh size	430.57 +/- 255.35	408.93 +/- 194.02	387.79 +/- 176.17	435.71 +/- 201.67	0.324
Rectus muscle violation	25 (21.2)	71 (25.7)	35 (20.7)	53 (33.8)	0.032
Parastomal hernia	5 (4.2)	25 (9.1)	11 (6.5)	27 (17.2)	0.001
Prior abdominal surgery	112 (94.9)	263 (95.3)	168 (99.4)	151 (96.2)	0.105
Prior hernia repair	29 (24.6)	60 (21.7)	45 (26.6)	44 (28)	0.463
Number of prior abdominal surgeries					0.328
0	6 (5.1)	13 (4.7)	2 (1.2)	6 (3.8)	
1	40 (33.9)	87 (31.5)	50 (29.6)	43 (27.4)	
2	35 (29.7)	97 (35.1)	51 (30.2)	53 (33.8)	
3	37 (31.4)	79 (28.6)	66 (39.1)	55 (35)	
Number of prior hernia repairs					0.496
0	90 (76.3)	215 (77.9)	125 (74)	113 (72)	
1	21 (17.8)	47 (17)	36 (21.3)	32 (20.4)	
2	5 (4.2)	8 (2.9)	8 (4.7)	10 (6.4)	
3	2 (1.7)	6 (2.2)	0 (0)	2 (1.3)	
Indication for repair					0.165
Extirpative defect	40 (33.9)	84 (30.4)	37 (21.9)	33 (21)	
Initial hernia	60 (50.8)	160 (58)	110 (65.1)	100 (63.7)	
Recurrent hernia	13 (11)	23 (8.3)	18 (10.7)	20 (12.7)	

© 2022 Hassan AM et al. *JAMA Network Open.*

Other	5 (4.2)	9 (3.3)	4 (2.4)	4 (2.5)	
Defect width					0.209
<=15 cm	79 (66.9)	194 (70.3)	117 (69.2)	97 (61.8)	
>15 cm	39 (33.1)	82 (29.7)	52 (30.8)	60 (38.2)	
Component separation	61 (51.7)	185 (67)	114 (67.5)	97 (61.8)	0.019
unilateral	28 (23.7)	57 (20.7)	34 (20.1)	23 (14.6)	
bilateral	33 (28)	128 (46.4)	80 (47.3)	74 (47.1)	
Bridged repair	11 (9.3)	32 (11.6)	14 (8.3)	23 (14.6)	0.283
Panniculectomy	23 (19.5)	82 (29.7)	95 (56.2)	61 (38.9)	<0.001

*43 patients (6%) had missing defect size values, 8 patients had missing mesh size values.

© 2022 Hassan AM et al. *JAMA Network Open.*

eTable 3. Reasons for Reoperation in the Cases Operated on by Fellows

Patient	Indication for AWR	Timing of Reoperation	Reoperation due to SSO/SSI?	Reason for reoperation	Senior surgeon scrubbed?
1	Pancreatectomy and Whipple procedure	4 months	Y	Seroma & abscess incision and drainage	Y
2	Incarcerated ventral hernia	2 months	Y	Abscess incision and drainage	N
3	Small bowl resection and ileostomy	7 months	Y	Ileostomy reversal due to anastomotic leakage	N
4	Appendiceal adenocarcinoma resection	8 years	N	Tumor recurrence	N/A*
5	salpingo-oophorectomy	2 weeks	Y	Abscess incision and drainage	N
6	Invasive bladder carcinoma	1 month	Y	Wound dehiscence	N
7	Gastroesophageal junction adenocarcinoma	1 week	Y	Wound dehiscence	Y
8	Radical nephrectomy	4 days	Y	Subcutaneous hematoma evacuation	N
9	Abdominal wall sarcoma	2 years	N	Hernia recurrence	N/A*
10	Invasive bladder carcinoma	8 months	Y	Abscess incision and drainage	N/A*
11	Metastatic colorectal carcinoma	6 months	Y	Abscess incision and drainage	N/A*
12	Radical nephrectomy	2 months	Y	Abscess incision and drainage	N
13	Metastatic colorectal carcinoma	1 month	Y	Abscess incision and drainage	Y

AWR, abdominal wall reconstruction, SSO, surgical site occurrences, SSI, surgical site infections. * reoperation occurred outside of fellowship time frame.

eTable 4. Univariate and Multivariable Hierarchical Logistic Regression Model of 30-Day Readmission

	Univariate model		Multivariable model	
**Variables**	OR (95%CI)	p	OR (95%CI)	p
Academic rank				
Fellow	Ref		Ref	
Assistant professor	0.80 (0.36 - 1.76)	0.577	0.62 (0.26 - 1.46)	0.273
Associate professor	1.14 (0.49 - 2.69)	0.759	0.64 (0.22 - 1.85)	0.413
Senior-level professor	0.87 (0.33 - 2.28)	0.775	0.41 (0.13 - 1.27)	0.122
Wound Classification				
Clean				
Clean-contaminated/contaminated/infected	3.02 (1.70 - 5.39)	<0.001	2.16 (1.16 - 3.99)	0.015
Age, y	1.00 (0.98 - 1.02)	0.778		
Sex, male	0.98 (0.61 - 1.58)	0.941		
Body mass index	1.01 (0.97 - 1.05)	0.611		
Obesity	0.87 (0.54 - 1.40)	0.558		
Tobacco use	0.29 (0.07 - 1.22)	0.091		
Any comorbidity	1.24 (0.74 - 2.08)	0.407		
Coronary artery disease	1.74 (0.83 - 3.65)	0.143		
Diabetes mellitus	1.30 (0.74 - 2.29)	0.353		
Pulmonary disease	1.56 (0.75 - 3.24)	0.237		
Renal disease	0.87 (0.36 - 2.13)	0.768		
Preoperative radiotherapy	1.13 (0.66 - 1.93)	0.653		
Preoperative chemotherapy	1.25 (0.75 - 2.09)	0.388		
ASA status				
2	Ref			
3, 4	2.33 (0.82 - 6.62)	0.112		
Rectus muscle violation	2.10 (1.28 - 3.45)	0.004	1.57 (0.92 - 2.68)	0.097
Parastomal hernia	1.74 (0.85 - 3.54)	0.129		
Prior abdominal surgery (yes vs. no)	3.65 (0.47 - 28.1)	0.213		
Prior hernia repair (yes vs. no)	0.77 (0.43 - 1.38)	0.379		
Indication for AWR (primary hernia vs. extirpative defect)	0.85 (0.50 - 1.44)	0.550		
Defect width, by 5 cm	0.82 (0.69 - 0.97)	0.018		
Defect length, by 5 cm	1.00 (0.81 - 1.23)	0.967		
Defect size, by 50 cm^2	0.92 (0.84 - 1.01)	0.069	0.92 (0.86 - 1.00)	0.050
Defect width >15 cm	0.52 (0.29 - 0.95)	0.034		
Component separation (yes vs. no)	1.04 (0.62 - 1.73)	0.893		
Bridged repair	1.65 (0.84 - 3.21)	0.144		
Panniculectomy	0.77 (0.43 - 1.37)	0.373		
Mesh width, by 5 cm	1.49 (1.15 - 1.93)	0.003		
Mesh length, by 5 cm	1.49 (1.26 - 1.78)	<0.001		
Mesh size, by 50 cm^2	1.12 (1.07 - 1.18)	<0.001	1.12 (1.06 - 1.19)	<0.001

OR, odds ratio; CI, confidence interval; ASA, American Society of Anesthesiologists; AWR, abdominal wall reconstruction.

© 2022 Hassan AM et al. *JAMA Network Open.*
